# Complex traces: Examining morbidity and mortality among 19th Century migrants to South Australia using a Complex Adaptive Systems framework

**DOI:** 10.1371/journal.pone.0320268

**Published:** 2025-07-17

**Authors:** Angela Gurr, Matthew Brook O’Donnell, Alan Henry Brook

**Affiliations:** 1 Adelaide Dental School, University of Adelaide, Adelaide, South Australia, Australia; 2 Annenberg School for Communication, University of Pennsylvania, Walnut, Pennsylvania, United States of America; University of North Carolina at Greensboro, UNITED STATES OF AMERICA

## Abstract

In multidisciplinary research interpreting interactions between diverse data sources requires a Complexity approach. A Complex Adaptive Systems (CAS) framework allows the relationships of multiple factors to be explored and may provide a more holistic and nuanced understanding. This study is innovative in explaining the potential benefits in a CAS approach to combining bioarchaeological and historical data when examining a rare archaeological skeletal sample of early migrants to South Australia (SA). Macroscopic, radiographic and micro-CT methods were used for the analysis of the skeletal remains of a group of 19^th^ century migrants buried in an unmarked area of St Mary’s Anglican Church Cemetery. The relevant historical records explored were from British emigrant ships to SA (1836–1885 CE) and the Church burial records (1847–1885 CE). Evidence of poor oral and general health was present in the skeletal material. Dental developmental defects indicated health insults in early life. Pathological manifestations in bone were compatible with joint and infectious diseases, and metabolic deficiencies. Historical documents recorded that the voyages to SA were challenging, with some ships experiencing a high death rate. Diseases, e.g., measles and scarlet fever, and diarrhoea were frequently recorded as causes of death at sea for both non-adults and adults. In the Colony, burial records showed similar causes of death for non-adults, but for adults, accidents and tuberculosis were often reported. The CAS approach provided insights beyond those from analysis of the individual sources. It increased understanding of emergent, non-predicted outcomes that resulted from interactions between multiple factors, the impact of fluctuating economy, political instability and ideological pressures, on the health of migrants. The CAS framework is a valuable methodology for interpreting health patterns and can be further developed including for a range of historical and contemporary health contexts.

## Introduction

### A Complex Adaptive Systems approach

**Fig 1:**
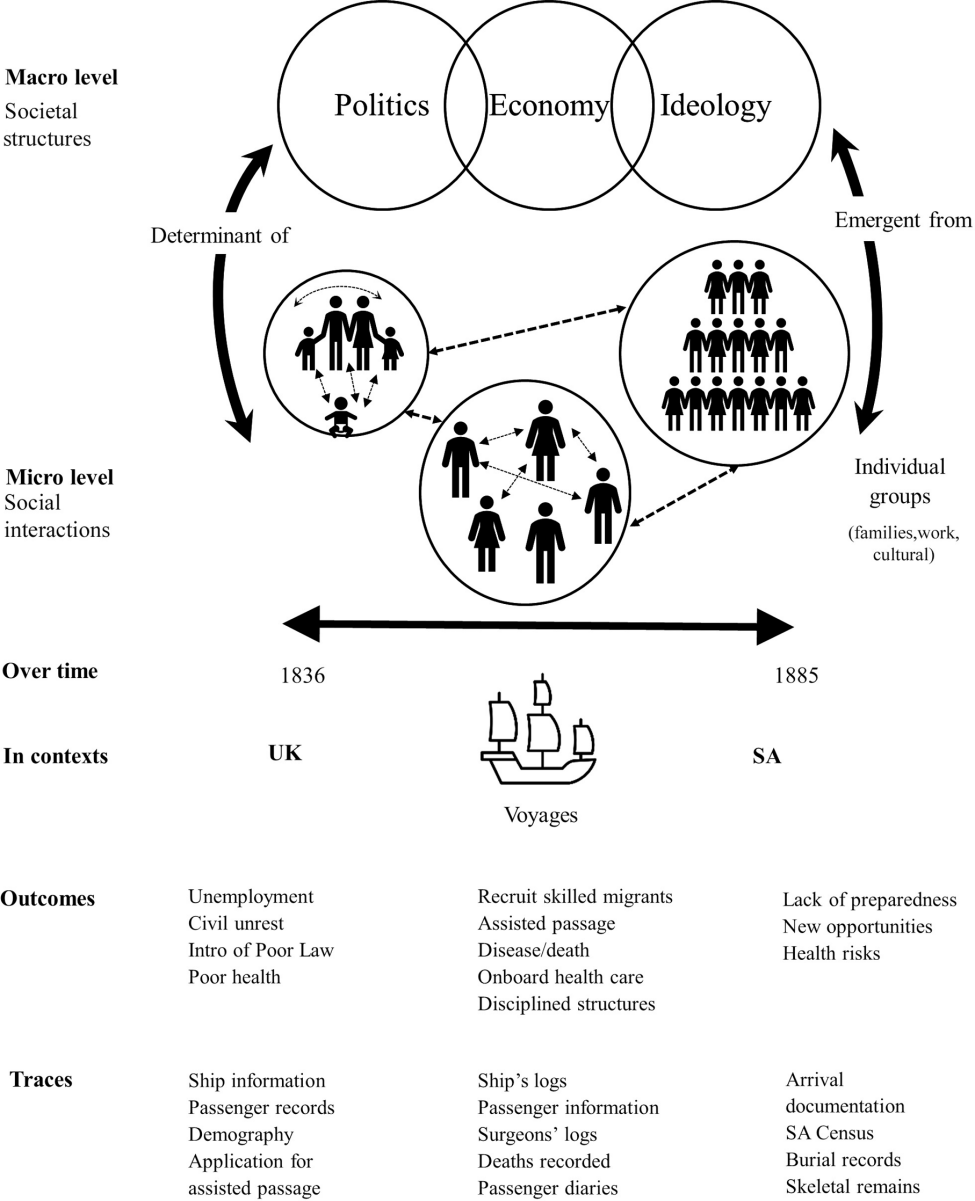
The characteristics of a Complex Adaptive System (CAS). Interactions occur on multiple levels and timescales and give rise to emergent properties and outcomes that cannot be predicted or anticipated from the individual parts.

Multidisciplinary research that integrates and interprets different types of data from diverse sources can be challenging. These challenges are seen across a range of scientific and other disciplines. Methods, such as the Complex Adaptive Systems (CAS) framework approach, have been developed to address them [1] and have been utilised in different research areas including biological development, social science and public health medicine [2–8]. The Complex Adaptive System has been described as “a group of semi-autonomous agents who interact in independent ways to produce system-wide patterns, such that those patterns then influence behaviour of the agents” [9,10] (Fig 1). The application of this approach, particularly within medical domains, to analyse findings from varied sources and investigate their relationships and interactions may provide new insights and a more holistic understanding of the data [2,4,7].

The characteristics of a Complex Adaptive System (CAS) are seen with the formation, development and ongoing functions of a social group, such as a new migrant colony [7]. Key components of a social CAS framework include interactions at multiple levels, between individuals such as passengers on a voyage, small groups, families, neighbours, companies and government structures in networks. These micro-level social interactions occur on multiple timescales and give rise to emergent properties (macro-level structures) and outcomes that cannot be predicted or anticipated from the individual parts (see top section of Fig 1). It is, therefore, a dynamic system that can be observed *over time* (e.g., 1836–1885), *across contexts* for example from British cities, to voyages at sea, and a colony such as South Australia (SA) , and in terms of *specific outcomes* such as poverty, migration, morbidity and mortality at sea and in establishing colony. These can be analysed in particular *data traces*, e.g., historical records for voyage passenger lists, deaths at sea, burial register and skeletal remains (see lower section of Fig 1). For instance, on a specific voyage from the United Kingdom (UK) to SA, an outbreak of an infectious disease might occur (environmental) and impact passengers in different ways based on age, their baseline health (genetics) and that of their parents (epigenetics). Some may not survive this insult on their health while others recovered and become members of the emerging colony, but carried life-long effects that may then have impacted their offspring.

The value of this perspective is that it can be used to guide the selection, organisation, analysis and interpretation of data sources and may provide a fuller context for these findings, enhancing understanding of individuals and groups studied. In Fig 1, moving from the bottom of the diagram upwards, shows data traces, which partially capture specific outcomes, that are occurring in particular contexts across specific times. Another example of the CAS approach would be data collected from the examination of human skeletal remains which provides evidence of dental developmental defects. The presence of these lesions shows that the individual suffered and survived one or more serious health insult in childhood or young adulthood (during dental development). This health event could have taken place during their voyage to the colony. Documentary data on the frequency of voyages, number of on-board fatalities, and records of causes of death during the likely period covering their childhood provide contexts for the necessary interactions that could give rise to the emergent dental defects.

This study investigates multiple sources of data including findings from the examination of archaeologically excavated human skeletal remains and diverse historical records, to understand the challenges faced by early migrants during the voyage and after arrival in the new colony of South Australia. The complex traces of the individual, family, group or organisation investigated here are for the period of 1836–1885 CE. These dates cover the initial period of *establishment* (1836–1848 CE), followed by *settlement* of the province (1849–1866 CE). The colony *matured* during the subsequent years (1867–1885 CE).

#### Background for the new colony of South Australia.

The American Revolution and independence from British rule (1773–1775/6) had caused political and commercial instability in Britain, arising from the loss of those colonies [11–13]. In Europe, the French Revolution of 1789–1794, brought civil unrest, social upheaval, and radical political changes [14,15], while the war with Napoleon had required substantial capital and resources [16,17]. The British aristocracy and political establishment were now greatly sensitized to the dangers of revolution by an oppressed populace. This was exemplified by the excessive use of force against a peaceful public protest that resulted in the Peterloo massacre (1819) [18].

The underpinning concepts and the formal establishment of the Provence of South Australia are well documented. Edward Gibbon Wakefield’s theoretical publication in 1829 proposed a systematic approach to establishing new colonies [19–21]. In these, rather than transporting convicts, voluntary emigration would be encouraged, and the British class system could be reproduced. Such new colonies could offer fresh opportunities for those affected by the gross overcrowding in the cities and towns of Britain and the greatly increased inequalities resulting from both international and national events.

The migration of workers from rural areas of Britain to urban industrial centres from approximately 1760–1850 followed the introduction of mass manufacturing of goods in factories using machinery [22–24]. Influxes of people in towns and cities without the infrastructure to cope produced overcrowding, unsanitary conditions, and the spread of disease [23–25]. Air and water pollution from factories adjacent to the workers’ housing also affected the health of the surrounding population [25–27].

During this period, the coast and rivers of South Australia were being explored by Matthew Flinders (1801–1803) [28,29], Charles Sturt (1828–1831) [30], and Collette Barker (1831) [31]. This region was seen as highly suitable for a new colony. With commercial backing in place from the formation of the South Australian Company [32,33], the British Parliament passed the South Australian Colonisation Act (1834) [34]. An emigration fund, suggested by Wakefield (1829) [21], for the conveyance of healthy young migrants and their families to a new colony was planned and the ‘assisted passage’ scheme, sponsored by the Colonial Commissioners became available for migrants who were able to comply with the selection criteria [20,34–38].

The first emigration ships for South Australia left Britain in 1836 [39–41]. These ships often carried different classes of passengers in cabin, intermediate and steerage/ assisted passage categories. There was a Superintendent Surgeon to attend to the health of the migrants during the many months at seas. Section 9 of the Passengers Act of 1835 [42] outlines the requirements for medical provisions onboard any ship conveying more than 100 people. It states that “some person duly authorised by law to practise in the UK as a Physician or Apothecary,…taking with him a Medicine Chest and a proper supply of medicine, and instruments…”. This position was independent from the ship’s crew, the Surgeon being employed by the Colonial Commissioner. The logs kept by these Surgeons provide a valuable but under explored data source of the health of the migrants during the voyage.

Arrival in the new Province of South Australia after a journey of up to 120 days did not end the challenges faced by the new migrant settlers. General environmental factors such as insufficient employment, a fluctuating economy and political instability [38], could have influenced their economic status and health as well as the eventual location of their burial.

While these historical events and decisions provide the necessary context for the migrant colony under investigation, viewing their interactions and the emergent outcomes they produced from a CAS perspective adds considerable value. There were short, medium and long-term outcomes from the interactions of the various decisions and actions, driven by ideological, economic, and political factors that were not, and likely could not have been, predicted at the outset. These outcomes could be either positive and negative and affect different individuals in different ways, the outcome in large part relating to individual resilience and the context. Therefore, the aim of this study is to explore if there are benefits in a CAS approach to combining bioarchaeological and historical data when analysing a rare skeletal sample of migrant settlers to SA.

## Materials and methods

### Data sources

All data sources used are from time periods within the Common Era (CE). A summary of the sources used in this study are given in Table 1.

**Table 1:** A summary of the data sources investigated in this study with date range of source.

Data Source		Type of Data	Date Range
**1**	**Bioarchaeological** St Mary’s Cemetery Human skeletal remains	Biological data from: Macroscopic & Radiographic examinations, Micro-CT scanning	1847-1927 CE
**2**	**Historical Documents** Ships records, Government records & Ship’s Surgeon’s logs:	Documentary	1836-1885 CE
**3**	**Historical Documents** St Mary’s Church Burial Records	Documentary	1847-1885CE

### 1. Bioarchaeological investigation of human skeletal remains

#### Ethics.

St Mary’s Anglican Church requested the excavation of the unmarked section of the cemetery and approved the study of this rare historical skeletal sample. Flinders University Social and Behavioural Research Ethics Committee provided ethics approval (SBREC Project number 8169). As this sample consisted of historical skeletal material of individuals whose identities are not known, there was no possibility or requirement to obtain informed consent.

#### Skeletal sample.

The skeletal remains of 70 individuals,19 adults and 51 non-adult migrant settlers to South Australia, were excavated from the unmarked section of St Mary’s Anglican Church Cemetery, near Adelaide, in 2000 [43]. From this total, 40 individuals (18 adults and 22 non-adults) had dentitions.

Burials in the unmarked section of the cemetery took place between 1847–1927. Previous studies have shown that most of these interments took place during the first decades after the establishment of the colony in 1836 [44–47]. The skeletal remains were accessed for research purposes from the 5^th^ of January 2021–31^st^ of November 2023, and between 22^nd^ January and 25^th^ July of 2024.

Data were derived from the macroscopic examination of the teeth and bones of the St Mary’s sample, and the radiographic examination and micro-CT scanning of the dentitions [45–47]. Details of the scoring systems, and standards followed for estimation of age range, determination of biological sex, and the identification of pathological manifestation of disease are presented in Table 2 and Table 3. These tables also include the criteria for the identification, and the systems followed to categorise tooth wear and/or pathologies related to oral health, such as evidence of caries, and periodontal disease. The scoring criteria of dental developmental defects (e.g., enamel hypoplastic defects and interglobular dentine) are also available in Tables 2 and 3.

**Table 2:** Categories used for scoring dental and alveolar bone health.

DENTAL Sample (N = 40)					
	**Classification**	**Criteria for Identification**	**Method**	**System/s for Scoring**	**Source** **Reference**
**Dental Inventory**	Total number of deciduous & or permanent teeth present	Ante-mortem tooth loss: evidence of alveolar bone healing. Post-mortem tooth loss: no evidence of healing	Macroscopic, Radiographic, Micro-CT	a) Tooth type/s present &identified using the FDI notation system b) Location of absent tooth & alveolar bone status including healed/ healing or unhealed, i.e., open socket with no tooth present	[51–59]
**Dental** **age range** **(0–23.5 years)**	Based on tooth development & eruption pattern	Stage of development- i.e., erupted, semi erupted & developing at the time of death within the alveolar bone/s for each tooth type	Macroscopic, Radiographic, Micro-CT	The London Atlas of tooth eruption & development (0–23.5 years)	[60–62]
**Tooth wear**	Loss of tooth structure due to wear	Loss of enamel &/or exposure of dentine due to attrition/ abrasion/ erosion	Macroscopic	Select category of tooth wear from Molnar’s (1971) visual criteria chart.	[63]
**Carious lesions** **(decay)**	Decay of the tooth structure	1) a) Decay present on enamel surface only, b) involving enamel & dentine, c) decay involving the enamel dentine & the pulp. 2) Identify changes in radiolucency/ density of the tooth	Macroscopic, Radiographic, Micro-CT	a) Tooth type affected b) Location of carious lesion in relation to the CEJ c) ICDAS/ICCMS category of radiolucency- using dental radiographs & DRRs. Select category from a visual chart	[64]
**Periodontal** **disease**	Morphological changes in the structure of the alveolar bone margins	Alveolar bone loss. Changes to the margins of the alveolar bone supporting the molars (posterior teeth)	Macroscopic	a) Measurement from the CEJ to the crest of the alveolar bone on the midline of the crown surface (labial/buccal & lingual/palatal) b) Alveolar bone status: Graded 0–4	[65–67]
**Enamel** **Hypoplastic** **defects** **(EH)**	A dental developmental defect of enamel where some enamel has not formed	Grooves, pits or areas of defects in the surfaces of the enamel due to early cessation of enamel matrix deposition during development	Macroscopic, Micro-CT	FDI – DDE & EDI indices used a) Type of EH defect/s, b) Number of EH defects on the enamel surface, c) Location of EH defect/s – measurement of the distance of the defect/s in relation to the CEJ.	[68–71]
**Interglobular Dentine (IGD)**	A dental developmental defect of dentine	Changes in density of the dentine structure due to mineralisation defects	Micro-CT	Presence of IGD as Yes/No	[72,73]

**NOTES:** FDI = Fédération Dentaire Internationale/ World Dental Federation Notation System, DDE = Developmental Defects in Enamel, EDI = Enamel Defect Index, CEJ = Cemento-Enamel Junction of the tooth, ICCMS = International Caries Classification & Management System, DRRs = Digitally Reconstructed Radiographs.

**Table 3:** Categories used for scoring skeletal health.

SKELETAL Sample (N = 70)					
	**Classification**	**Criteria for** **Identification**	**Method** **of** **Examination**	**System/s for Scoring**	**Source** **Reference**
**Biological Sex**	Physical characteristics attributed to a biological male or female	Morphological features ascribed to sexual dimorphic changes in the skeleton.	Macroscopic	Morphological features: a) Pelvic (Os Coxae) & b) Skull (cranial)	[74–76]
**Spinal & Extraspinal** Joint Disease	Pathological & or degenerative changes to bony joints	Morphological features associated with 1) Osteoarthritis affecting synovial joints, 2) Degenerative joint disease	Macroscopic	Morphological features: Degree of expression: a) Lipping marginal to the articular surface, b) Porosity of the surface, c) Eburnation d) Osteophytes, e) Syndesmophytes/enthesophytes e) Schmorl’s nodes.	[75,77,78]
**Metabolic disturbances/ deficiencies**	Conditions affecting the balance of chemical &/ or metabolic processes of the body	Pathological manifestations on the bone/s ascribed to insufficient nutritional intake, i.e., a deficiency of Vitamin C, Vitamin D, & or Iron	Macroscopic	†Examples of pathological manifestations for Vit C &/or Vit D, & /or Iron deficiency include: abnormal porosity in the bone cortex, new bone growth, bending & distortion of bones etc.	[79–88]
**Infection**	Conditions caused by microorganisms – such as bacteria*	Pathological manifestations on the bone/s attributed to bacterial infection	Macroscopic	^†^Pathological features associated with: a) Tuberculosis, b) Leprosy c) Treponemal disease	[43,89–95]
**Trauma**	Traumatic injuries to the bones of the skeleton	Damage to, or alteration/ abnormal displacement of the bone/s caused by external forces	Macroscopic	^†^ Features associated with: a) Fractures, blunt force injury, &/or dislocation. b) Evidence of bone healing, &/ or infection.	[75,96,97]

**Notes**:

† The list of pathological features for some of the conditions mentioned in Table 3 is too long to include here.. Please see cited sources for full details. * Microorganism such as viruses, fungi, and parasites etc. may also cause infections however, bacteria are a common cause [89,90]

Investigation of dental developmental defects and pathologies associated with poor oral hygiene, as well as pathological manifestations and/ or traumatic injuries on the bone, provides valuable evidence of past health challenges in the life histories of these individuals from St Mary’s Cemetery.

Information of the settings for the radiographic and the Micro-CT scanning systems used, e.g., Small Volume Micro-CT system – Bruker SkyScan 1276 [48], and the Nikon XT H 225 ST – Large Volume Micro-CT system [49] is published by Gurr et al., [46,50].

### Limitations of the Skeletal remains source

The skeletal remains investigated in this paper are a specific group of the local community of St Marys on the Sturt, South Australia, who at the time death could not afford the burial costs.The nature of this groups interment makes this sample is biased. For example, the demographic profile of this group as listed in the burial records, lacks individuals (adults and non-adults) from higher socioeconomic classes. Consideration is given to the Osteological Paradox when analysing the data, i.e., “effects of heterogeneous frailty and selective morality on health inferences in past populations” [98,99], and that pathological lesions seen on these skeletal remains are markers of disease and not of health. This is, however, currently the only South Australian human skeletal sample excavated from a colonial cemetery available for examination. While the individuals excavated from the unmarked section of St Mary’s Cemetery cannot represent the entire colonial population for this period, they are a rare and extremely valuable source of data that provides an insight and deeper understanding of some of the lived experiences. . There were limits regarding the extent to which information relating to the health of the excavated individuals could be collected from the skeletal remains as no data relating to conditions that affect the soft tissues of the body could be gained.

### 2. Ships Records - Historical documentation

This study investigates two types of historical ships records, the government records and the logs belonging to Surgeon Superintendant of the ship.. Firstly, the available documents relating to the emigrant ships that carried more than ten passengers from the United Kingdom (UK) to South Australia (SA) from 1836 to 1885 CE [100,101], were investigated to provide information on the number of passengers and the conditions on board the ships such as the Summary Report and the Certificate of Final Departure [100–102].

The documents completed by the Superintendent Surgeon assigned to each migrant ship from 1849 to 1865, as published in the South Australian Government Gazette [103], were used as they often recorded the cause of death for passengers who died at sea. The periods covered by these types of historical ships records differ slightly due to the availability of the data sources (Table 1).

### Limitations of Ships Records

The data used and the findings of this study relating to migrant deaths at seas could be skewed as many of the emigrant ships from the UK to SA during the study period (1836–1885 CE) had no fatalities. However, the length of the journey, condition on route such as overcrowding on board ship and or the weather on route meant that there was loss of life on many voyages. Other limitations of historical records include variability in the details recorded by the Superintendent Surgeon, the medical terminology used, and the readability of the text. Historical sources are rarely a complete and comprehensive record and may contain bias due to both individual perspective and prejudices of the broader political and social culture. Damage to and loss of records over time is also a problem. For data relating to the ‘causes of death’ at sea used in this study, this was limited to the period of 1849–1865 CE, due to the availability of published sources.

### 3. St Mary's Church Records - Historical documentation

Parish burial records for St Mary’s Anglican Church Cemetery, SA, began in 1847 [104]. To reflect the temporal range used for the records belonging to the emigrant ships(i.e., 1836–1885 CE), the burial records have been analysed until 1885. These documents provide insights into mortality during the early decades of the colony for a specific group of migrant settlers. These documents show that N = 143 individuals were interred *either* in i) the unmarked section of St Mary’s Cemetery – which was often listed as the ‘free ground’, ‘common ground’, or ‘unleased ground’, *or* ii) the location of their burial within this cemetery was not recorded, *or* iii) the location of their burial is unknown due to damage to the church records.

### Limitations of Church Records

A fire at St Mary’s Anglican Church in 1953 CE destroyed many historical records stored in the building [105,106]. This loss of data is a limitation that would have affected the data relating to burials in the unmarked section of the church cemetery. The available burial records list more individuals than were excavated from the unmarked area which means there may be some people listed in the burial records that were not excavated and are still buried in the adjacent unmarked plots.

## Results

Table 4 provides an overview of the analyses and types of results obtained from the three data sources. The key variables from each source and how they were prepared for analysis and modelling are summarised.

**Table 4:** A summary of the key variables from each source and how they were analysed and prepared for modelling.

Data Source		Unit of Analysis	Key Variables	Analysis & Modelling
**1**	**Bioarcaeological** St Mary’s Cemetery - Human skeletal remains	Individual	Age, biological sex, dental development defects, tooth wear, caries, periodontal disease, antemortem tooth loss. Skeletal pathologies, including vertebrae pathologies, metabolic disturbances, infections & trauma	Age group, descriptive observations, potential causes & future health implications
**2**	**Historical Documents** Ships records, Government records, Ship’s Surgeon’s logs	Voyage Passenger	Date, count data by passenger type, number of deaths. Year of death, age, cause of death.	Aggregation by period & time series/trend analysis Aggregation by cause of death & time series/trends analysis
**3**	**Historical Documents** St Mary’s Church burial records	Individual	Estimated age, Cause of death	Grouped counts & aggregation by period

### 1. Bioarchaeological investigation of human skeletal remains

The demographic profile, with the estimation of age range and determination of biological sex, for the individuals excavated from the unmarked section of St Mary’s Cemetery is presented in Table 5. It shows that many of these individuals were infants and non-adults. The identity of the 70 individuals is not known due to the lack of identifying markers such as gravestones.

**Table 5:** 1847-1927 CE– Demographic profile for the individuals excavated from the unmarked section of St Mary’s Cemetery with the number of non-adults and adults within each age range category and biological sex.

Age range at death (years)	Biological Sex			Totals N=
	Undetermined	Female	Male	
**Infants of unknown age**	21	N/A	N/A	21
**0-3 months**	6	N/A	N/A	6
**4-11 months**	5	N/A	N/A	5
**1–2**	9	N/A	N/A	9
**3–4**	2	N/A	N/A	2
**5–9**	3	N/A	N/A	3
**10–14**	4	N/A	N/A	4
**15–19**	0	1	0	1
**20–29**	0	2	0	1
**30–39**	0	3	2	5
**40–49**	0	3	8	11
**50–59+**	0	0	1	1
**Total – non-adults**	**51**	**0**	**0**	**51**
**Total – adults**	**0**	**8**	**11**	**19**
**Grand total** **Number of individuals**				**70**

**Fig 2:**
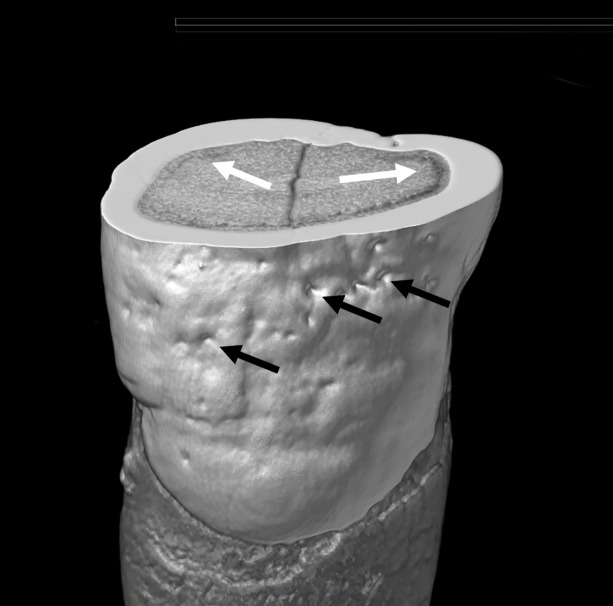
Small Volume Micro-CT – An image of a human tooth with developmental defects. This transverse (cross-sectional) view shows the exterior and interior surfaces of a permanent upper incisor. Pitted defects in the enamel (black arrows) and areas of interglobular dentine (white arrows), are highlighted.

The multiple bioarchaeological techniques used for the analyses of the skeletons and dentition (Tables 2 and 3) i.e., macroscopic, radiographic, and large and small volume micro-CT scanning, identified evidence of poor oral and general health conditions, as well as signs of earlier survived health insults in the form of dental developmental defects (Fig 2).

Pathological manifestations and or changes to the teeth and bones due to disease and or deficiencies and or trauma observed in the St Mary’s individuals are listed in Table 6. A full list of the conditions investigated is given in Tables 2 and 3. The criteria for identification for the dental and skeletal conditions with the scoring systems used and citations for these systems are also found in Tables 2 and 3.

**Table 6:** 1847-1927 CE – Health conditions and defects observed on teeth and bones of the St Mary’s Cemetery individuals, with the probable cause and future health implications.

Condition/s	Observed Evidence	No. & % of total affected individuals N = 70	Cause & Implication for Health
**Oral** **Health** (N = 40) [46]	Carious lesions	17 adults 4 non-adults 53%	Extensive caries (decay) is associated with a diet high in carbohydrates, particularly sugars. Decay of the enamel surface leads to exposure of the dentine & pulp. This will cause pain/infection, & eventual tooth loss.
	Periapical lesion/s	1 non-adult 3%	A peri-apical lesion seen on a radiograph may arise from a cyst, a dental granuloma, or more commonly a dental abscess [65,107]. The lesion observed indicated an abscess as it was associated with a large carious lesion that involved the enamel, dentine & pulp. The caries progresses to infection of the pulp, which spreads to the periapical tissues and abscess formation.
	Calculus	11 adults 28%	A build-up of calcified dental plaque can lead to gingivitis, periodontitis & periodontal disease.
	Periodontal disease	9 adults 23%	This condition leads to alveolar bone recession, & tooth loss, as well as increasing the possibility of co-morbidities such as cardiovascular disease or stroke. [108–112]
	Antemortem tooth loss (less than 20 permanent teeth in situ) [113]	13 adults 33%	A reduction in tooth number will affect the ability to eat certain food types and may reduce nutritional intake.
	Smoking – pipe notch	3 adults 8%	Smoking tobacco affects the oral mucosa and oral biome. It also increases the possibility of other co-morbidities affecting the body’s systemic health.
**Tooth Wear** abrasion/ attrition/ erosion [47] (N = 40)	Diet, health related, including coarse food, grinding teeth, and acidic drinks Gastro-Oesophageal Reflux Disease (GORD) [114–116]	14 adults (Category 5 +) [63] 35%	Tooth wear that exposes the dentine can cause pain, eventually exposing the pulp leading to infection & tooth loss.
**Dental Developmental Defects** [46] (N = 40)	Enamel hypoplastic defects	14 adults & 10 non-adults 60%	Serious insults to the body’s systemic health during dental development can cause cessation of tooth enamel matrix production leading to deficiencies in the enamel thickness. The macroscopic appearance is enamel hypoplasia.
	Interglobular dentine	1 adult & 1 non-adult 5%	Serious insults to the body’s systemic health during dental development, including vitamin D deficiency, can cause a reduction or cessation of the mineralisation of the dentine structure, leading to area with less density.
**Metabolic Deficiencies** [48] (N = 70)	Folic Acid – Vitamin B9 deficiency during pregnancy	5 adults 7%	Spina bifida occulta is the failure of neural tube to completely close. This condition can cause vulnerability in the posterior section of the sacrum
	Vitamin. C deficiency	1 non-adult 1%	This deficiency affects the quality & quantity collagen and can cause inflammation, bleeding, & pain. It is potentially fatal if chronic and severe.
	Iron deficiency	3 non-adults 4%	This deficiency affects the haemoglobin in the red blood cells. It causes fatigue and weakness due to a reduction in the oxygen reaching the brain & muscles
**Spinal & Extraspinal** **Joint** **Disease** [45] (N = 70)	Morphological changes – pathological & or degenerative 1) Osteoarthritis: eburnation of the joint surface 2) Degenerative disease: osteophytes, & Schmorl’s nodes	8 adults 11%	Changes in the bone morphology of the joints can be caused by repetitive movements such as hard physical labour over a long period of time. These pathological changes can reduce mobility, causing pain & eventual disability.
**Infectious Disease/s** [43,91–95] (N = 70)	Congenital syphilis (Treponema pallidum)	1 non-adult 1%	This infectious condition is passed from the mother to the child during pregnancy. Non-adults may display multiple signs for this condition involving soft tissues, bones and dentition.
	Tuberculosis (Mycobacterium tuberculosis)	1 non-adult 1%	This airborne infectious disease can affect the lungs and other tissues of the body including causing destructive lesions of the vertebral bodies & other bones causing pain, disability and death.
**Trauma** [47,77,98,99] (N = 70)	Damage to, or alteration/ abnormal displacement of the bone/s caused by external forces Perimortem trauma – No evidence of healing/infection/new bone growth	2 adults 3%	Traumatic injuries such as a fracture to one or more bones of the skeleton may cause severe pain, mobility or other related health issues or be fatal.
	Antemortem trauma – bone injury has healed and remodeled	3 adults 4%	

### 2. Ships records

#### Number of voyages and passengers over time −1836–1885 CE.

**Fig 3:**
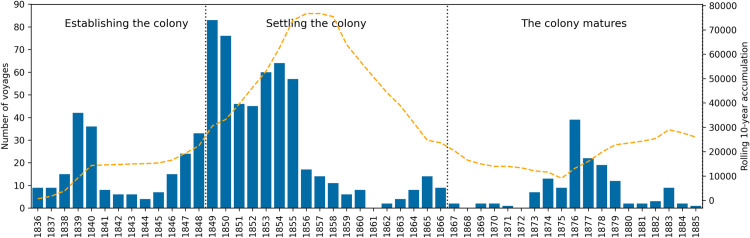
1836–1885 CE. Number and distribution of emigrant voyages, carrying more than ten passengers from the United Kingdom to South Australia, in relation to the year of arrival [100,101]. Cumulative addition of individuals to the colony shown in the orange dashed line using a 10-year rolling window.

In total there were 885 voyages carrying more than ten passengers from the UK to SA from 1836 to 1885. The number of ships arriving in SA each year are shown in Fig 3. The dashed line shows the accumulation of passengers from these voyages using a rolling 10-year window as a way of illustrating the impact of new arrivals in the growing colony over this time. There are no recorded emigrant ships arriving in SA for 1861, 1868 and 1872 CE and a single voyage in each of 1871 and 1885 [100,101]. The busiest years were: 1849 with 83 voyages, 1850 with 76 voyages, 1854 with 64 voyages, 1853 with 60 voyages, and 1855 with 57 voyages. These events all occurred within the second period (1849–1870) (Fig 3).

During the first period (1836–1848), in which the colony was being established, the average number of voyages per year was 16.5 (SD 13.0), driven by two key years 1839–1840 followed by a return to ten or less voyages per year until 1846. This is reflected in the flat accumulation line in Fig 3. The second period (1849–1866) saw the settling and stabilization of the colony with a high demand for workers in the initial period up until 1855. The average number of voyages per year during this period was 29.1 (SD 28.2). However, dividing this period into an early section (1849–1855), with an average of 61.6 (SD 14.2) voyages per year and a later section (1856–1866), with an average of 8.5 (SD 5.3), better captures the complex dynamics of the settling period.

The accumulation of new arrivals (over a ten-year period) peaks towards the middle of the second period (around 1857) fitting with communities becoming established and organised. The final section (1867–1885) sees the colony maturing and requiring fewer external workers to sustain growth and development. This is reflected in the lowest average number of voyages per year of 7.7 (SD 10.0).

#### 2.1. Number and Type of Mortality at Sea Over Time – 1836–1885.

**Fig 4:**
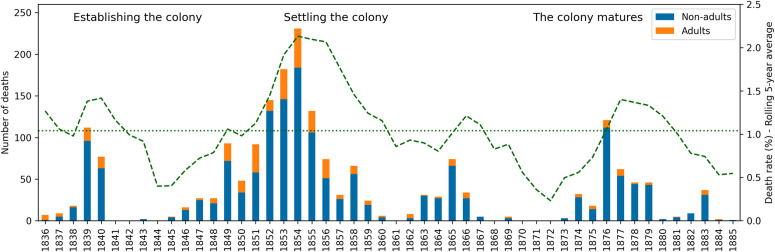
1836–1885 CE. Number of non-adult and adult deaths by year as recorded on emigrant voyages to South Australia from the United Kingdom with more than 10 passengers. [100,101]. Average death rate per year approximately 1% (dotted line) with 5-year average shown in dashed line.

There were many risks to the health of the 19^th^ century migrants bound for South Australia with some passengers dying during the long voyage. The number of non-adult and adult deaths at sea per year from 1836–1885, as recorded for British emigrant ships to South Australia is shown in Fig 4. Individuals who died during the voyage would have been buried at sea.

Table 7 provides the findings for the ships with high mortality rates on emigrant voyages from the UK to SA from 1836–1885 [100,101]. It presents a breakdown of the number of passengers and deaths on each ship, together with the percentage of fatalities recorded.

**Table 7:** 1836 −1885 CE. Emigrant Ships by year of departure with a high percentage of deaths at sea carrying more than ten passengers from the United Kingdom to South Australia [100,101].

Name of Ship	Year of Departure	Number of Passengers	Number of deaths	Death %
Prince Regent	1839	199	20	10%
Resource	1839	211	30	14%
Fairfield	1840	186	35	19%
Himalaya	1849	176	29	16%
Douglas	1850	125	21	17%
Shackamaxon	1852	696	65	9%
John Bunyan	1854	340	29	9%
James Fernie	1854	376	30	8%
Morning Star	1862	452	26	6%
Lochee	1877	524	26	5%

#### 2.2. Superintendent Surgeons’ Logs – Causes of Death at Sea – 1849–1865 CE.

**Fig 5:**
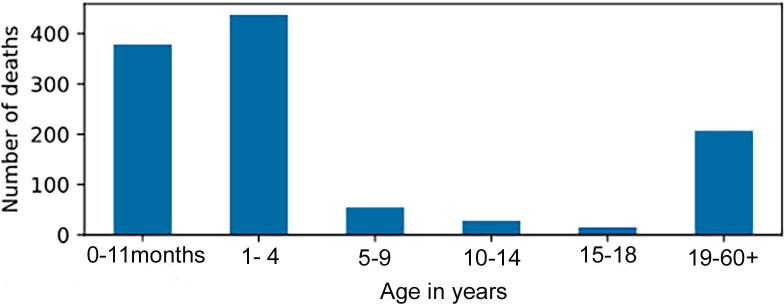
1849-1865 CE. Number of deaths by age range in years for passengers on board emigrant ships to South Australia from the United Kingdom [103].

Investigation of the Superintendent Surgeons’ logs for the period 1849–1865 provides the number of deaths at sea for the study period [103]. These data are divided into different age groups: 0–11 months, 1–4 years, 5–9 years, 10–14 years, 15–18 years and 19–50 + years. Non-adults under the age of four years were impacted the most during the voyage as highlighted in Fig 5.

The death of a passenger at sea (1849–1865) [103] could have been caused by multiple interacting factors including infectious and non-infectious conditions. Table 8 lists the frequently recorded non-infectious and infectious conditions for all age groups on emigrant voyages from the UK to SA during this period.

**Table 8:** 1849 −1865 CE. Non-infectious and infectious conditions recorded for all age groups on emigrant voyages from the United Kingdom to South Australia by the ship’s surgeon with a modern equivalent or explanation [54].

Commonly recorded causes of death at sea	
**As recorded by Ship’s Surgeon**	**Modern equivalent/ explanation [117]**
**Non-infectious conditions/symptoms and signs**	
Apoplexy	Sudden, severe stroke-like symptoms and signs.
Atrophy, Exhaustion, Inanition, Tabes	Physical weakness, wasting away of the body, failure to thrive. Exhausted condition that results from lack of food & water.
Convulsions	Seizures – abnormal violent & involuntary contractions of the muscles.
Found dead	Unknown cause of death.
Inflammation of bowels, or kidneys	Irritable Bowel Syndrome/ Inflammatory Bowel Disease, Nephritis – inflammation of the kidney caused by infection, degenerative process or vascular disease.
Marasmus	Chronic undernourishment/ energy deficiency – occurring in children & usually by deficiency in calories & protein intake. See also Tabes, exhaustion, inanition etc.
Mesenteric disease	Conditions related to the Mesentery (soft tissue supporting the intestines) such as narrowed/ blocked arteries to the small intestine.
Pneumonia	Acute disease – inflammation of the lungs, infiltration of alveoli & bronchioles with white blood cells & fibrinous exudate. Difficulty breathing, chest pain & reduced lung capacity. Often caused by an infection.
Premature birth, still born	Died before or soon after birth.
Teething	Death occurring during the period of teething – multiple causes.
Suicide	Intentionally & voluntarily causing one’s own death
Sunstroke	Heat exhaustion/ heat stroke -body is unable to regulate its temperature. Overheating – above 40°C, brain damage & organ failure.
**Infectious conditions/ symptoms**	
Bronchitis/ Croup	Inflammation of the airways leading to the lungs – bronchial tubes. Croup = virus that causes inflammation, oedema of the larynx, trachea & bronchi affects infants & young children.
Cholera	Bacterial disease caused by *Vibrio cholerae*. Usually spread through contaminated water leading to severe diarrhoea, vomiting & rapid dehydration.
Congestion/ effusion/ inflammation of the brain	Encephalitis, Meningitis – serious infections causing inflammation of the brain or tissues surrounding it
Consumption/ Phthisis	Tuberculosis – bacterial (*Mycobacterium tuberculosis*) infection of the lungs and other areas of the body. Characterised by fever, cough & difficulty breathing.
Diarrhoea, Dysentery	Gastrointestinal conditions – abnormally frequent intestinal evacuations – fluid stools. Severe diarrhoea passing mucus & blood often caused by an infection.
Fever/ low fever/ remittent fever	Increase in the body’s temperature above the normal range often caused by a viral or bacterial infection
Measles	Contagious viral infection caused by a member of the *Morbillivirus* genus in the *Paramyxoviridae* family. characterised by red circular spots
Pertussis/ Whooping cough	Infectious respiratory disease – especially of children- caused by a bacterium – convulsive spasmodic cough followed by a ‘crowing’ intake of breath.
Scarlatina	Contagious viral infection caused by *Group A streptococcal* bacteria. Characterised by a red rash, fever, inflammation of the nose, mouth & throat.
Tabes mesenterica	“Abdominal Tuberculosis” [118,119] in infants under one year of age. Characterised by emaciation, abdominal enlargement, & diarrhoea – affecting the mesenteric lymph nodes.
Typhoid fever	Intestinal inflammation caused by a bacterial infection -*Salmonella Typhi*, usually spread through contaminated food or water. Characterised by fever, diarrhoea, & headache.
Typhus	Bacterial infections –*Rickettsiae*. Spread through ectoparasites such as fleas, lice & ticks. Characterized by purple rash fever, chills, body aches, vomiting, delirium & seizures

Documentation for 142 emigrant ships that sailed from the UK to SA (1849–1865) [103] show that from the 89165 passengers recorded in voyage records during this period, 1141 deaths were logged. Therefore, around 1.3% or 1 of every 80 died before arrival. From this total number of deaths, 387 (34%) have no cause or explanation recorded. Analysis of these data is further complicated due to a variety of spellings and terms used to record the cause of death.

The most commonly documented causes of death for each age group on board of the 142 voyages [103] are given in Table 9. These are shown in the order of the frequency that they were recorded in the ships’ logs, e.g., numerous non-adults under one year of age died due to diarrhoea or dysentery followed by debility, convulsions and bronchitis. Table 10 breaks down this data further showing the number of deaths for each ‘cause’ and in which year/s had the highest number or a peak in the recorded deaths on emigrant voyages to South Australia from the United Kingdom from 1849–1865 [103].

**Table 9:** 1849-1865 CE. Causes of death at sea in order of most frequently recorded by age group on migrant ships from the United Kingdom to South Australia [103].

Age range (years)	Causes of death at sea in order of most frequently recorded by the ship’s surgeon				
**0-11 months**	Diarrhoea/ Dysentery	Wasting (*Atrophy, exhaustion, inanition, marasmus, tabes)	Convulsions	Debility	Typhoid Fever,
**1-4**	Diarrhoea/ Dysentery	Measles	Scarlatina	Convulsions	Pneumonia
**5-9**	Cholera	Marasmus	Bronchitis	Scarlatina	Measles
**10-14**	Cholera	Scarlatina	Diarrhoea	Debility	Phthisis
**15-18**	Dysentery	Phthisis	Cholera	Diarrhoea	No record
**19-60+**	Fever	Diarrhoea/ Dysentery	Cholera	Phthisis, Bronchitis/ Pneumonia	Typhus

Note: See Tables 8 for explanations or definition of conditions. *All of these conditions are associated with physical weakness, wasting away of the body, or failure to thrive.

**Table 10:** 1849-1865 CE. Top 15 causes of death on emigrant voyages to South Australia from the United Kingdom from 1849-1865 (Total Deaths: 1141) [103].

Cause of death	Count	Peak of	In year
Diarrhoea	155	42	1854
Fever	71	30	1854
Convulsions	41	20	1854
Debility	40	10	1854
Measles	38	10	1854
Marasmus	32	10	1855
Dysentery	29	11	1854
Cholera	29	29	1854
Bronchitis	24	6	1854
Typhoid fever	24	17	1854
Scarlatina	23	7	1863
Pneumonia	19	7	1855
Consumption	13	3	1854 & 1856
Croup	12	3	1855 & 1858
Atrophy	12	9	1854

### 3. Church burial records

**Fig 6:**
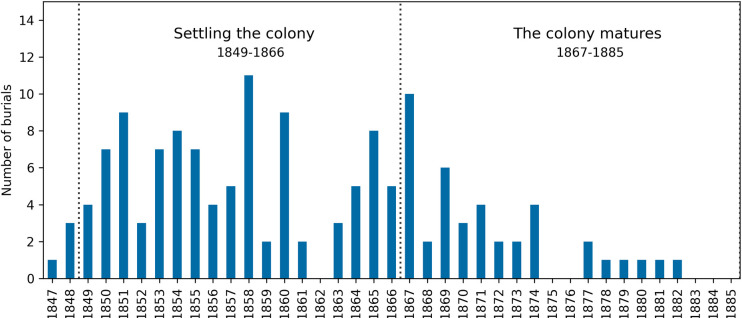
1847–1885 CE. St Mary’s Church Burial Records -Year of burial and number of individuals – (total N = 143) listed as interred in the unmarked section of St Mary’s Cemetery, South Australia, (or the location of their burial was not recorded, or is unknown due to damage to records) [43,104].

Burial records for St Mary’s Anglican Church, SA, show that a group of 143 individuals *could* have been interred in the unmarked ‘free ground’ area of the cemetery (see materials for details) [104].These burials, paid for by the SA government, fluctuated between 1847 and 1885 CE (Fig 6), with the majority of them taking place before the 1870s [45]. The number of interments in the unmarked section declined after this time (Fig 6). The highest number of deaths/burials for this group of 143 individuals where among the infants – age range 0–11 months (65/143), followed by the non-adults – age range 1–3 years (39/143) (Table 11) [43].

**Table 11:** 1847-1885 CE. St Mary’s Cemetery Records– Most frequently recorded causes of deaths with age ranges of the deceased [43].

Commonly recorded causes of death with the number of individuals affected							
	Infant 0-11 months N = 65	1-4 years N = 39	5-9 years N = 4	10-14 years N = 5	15-18 years N = 1	19-50+ years N = 29	Grand Totals N = 143
Accident	0	0	0	1	1	3	5
Affliction of the brain	0	4	0	0	0	2	6
Atrophy/ Debility/ Marasmus	10	3	0	0	0	0	13
Cancer of the womb	0	0	0	0	0	2	2
Causes not recorded	21	8	0	2	0	6	37
Convulsions	7	2	0	0	0	0	9
Dropsy	1	0	1	0	0	1	3
Dysentery/ Diarrhoea	8	8	0	0	0	0	16
Fever	0	0	1	0	0	2	3
General decay/ old age	0	0	0	0	0	2	2
Heart conditions	0	0	0	1	0	1	2
Liver or Kidney conditions	0	0	0	0	0	1	1
Mesenteric disease	3	1	0	0	0	0	4
Premature birth/ Still born	2	0	0	0	0	0	2
Pulmonary conditions (including: bronchitis, croup, phthisis, pneumonia, whooping cough)	2	3	1	0	1	6	13
Teething	5	5	0	0	0	0	10
Other conditions listed (Anasarca, Gastritis, trismus, thrush, obstruction of the bowel, spinal complaint etc.)	6	5	1	1	0	2	15

Note: See Table 8 for explanations or definition of conditions.

Records frequently listed diarrhoea and dysentery followed by atrophy as a cause of death for the St Mary’s infants and non-adults (Table 11). Whereas pulmonary conditions such as Phthisis (tuberculosis) were commonly listed as causes of death for the adults. Accidents caused the death of three adults from this group (Table 11) [43].For some individuals a cause of was not recorded (Table 11). This is especially true for the youngest age group.

## Discussion

### Integration of the multiple data sources

This paper investigated the interactions of data from three different sources. This integration of multiple data sources (Table 1) provided an insight into the morbidity and mortality for early migrants both at sea and in South Australia. The limitations and biases of each individual source indicate the value of the CAS approach (Fig 1) as it allows the combination of the sources to set up the likelihood of certain outcomes, e.g., evidence of dental developmental defects observed in the sample of St Mary’s Cemetery skeletal remains, based on prior incidents such as a wide scale outbreak of a serious disease on a voyage that affected children and young adults. For some passengers, health issues such as exposure to disease and/or by poor conditions on board the ship, such as contaminated food and water supplies could have increased their susceptibility to further health insults. Infectious diseases including measles and smallpox could have spread more quickly on-board emigrant ships due to the concentration of individuals in a confined space compared to on land.

The St Mary’s burial records and the information gained from the multi-methodological analyses of this specific group of skeletal remains represented a rare opportunity to investigate coherent and consistent data traces for these individuals. The individuals were all interred in the same area designated for those who did not have funds at the time of death to pay for a burial plot. These people were likely to have experienced the impact of the same emergent social structures and difficulties such as economic pressures and health challenges. The St Mary’s Cemetery sample is unique and by its nature biased. Therefore, it is not representative of the whole colony but of a coherent stratum during the study period (1836–1885 CE). However, the same trajectory of analysis from additional data traces, for instance if analysis of skeletons from higher societal strata buried in marked graves could potentially be carried out, if they became available in the future. Any clusters of emergent structures and outcomes in wealth and health predicted to have impacted their lives, time and manner of death may also be reflected in their skeletal and dental remains.

The findings for the combined data sources showed a general trend for high infant (0–11 months) and young non-adult mortality, both at sea during the migrants voyage and also after arrival and settlement during the early years of the colony. The young demographic profile of passenger deaths at sea (Fig 4), was reflected in the burial records associated with St Mary’s Church (Table 11). This documentary trend was confirmed by the high number of non-adults excavated from the unmarked section of St Mary’s Cemetery (Table 5). In the colony during the early period (1836–1848), rudimentary health care was available in the city [120], but for individuals living in communities away from these facilities reduced access to a doctor could prove fatal. The health of the younger age groups would have been impacted more than the adults due to their small body size and the reduction of the maternal antibodies combined with a still developing immune system. These factors would have reduced their ability to recover from the effects of disease such as gastrointestinal conditions and associated dehydration compared to an adult [121].

The background of the individuals excavated from the unmarked area of St Mary’s Cemetery and those listed in St Mary’s Burial records is unknown, but the majority of them are likely to have been born in Britain. Some may have had a low socioeconomic status, working in a factory or as labourers. Having survived the voyage to South Australia, they went on to settle and experience a new lifestyle. Many individuals thrived in the colony, seizing new opportunities, building commercial enterprises or substantial landholdings. However, the financial position of those buried in the unmarked section of St Mary’s Cemetery at the time of death and the evidence from their skeletal remains show that they did not prosper. . Rather the challenges of the new life, the volatility of the economy and the limitations of the infrastructure proved overwhelming.

### 1. Bioarchaeological investigation of St Mary’s Cemetery Sample

Poor oral health, with evidence of extensive carious lesions, periodontal disease, pipe smoking and widespread antemortem tooth loss, affected the majority of the adults in the St Mary’s skeletal sample (Table 6) [46]. Previous studies have shown that these findings are comparable to other contemporaneous agricultural and or industrial communities (Table 12) [46]. Poor oral health would have negatively influenced the individual’s general health status.

**Table 12:** Previously published findings for St Mary’s Cemetery dental sample and comparable contemporary skeletal samples from cemeteries in the United Kingdom, Australia and New Zealand.

	Cemetery			
Dental Condition [46]	St Mary’s (SA) 1847-1927 CE N = 40	Cross Bones (UK) 1800 −1853 CE N = 83	Cadia (NSW) 1864-1927 CE N = 109	St John’s (NZ) 1860-1926 CE N = 7
Tooth wear (moderate to heavy)	14/40 35%	No Data	No Data	7/7 100%
Caries	21/40 53%	44/83 53%	32/109 29%	6/7 86%
Periodontal disease	9/40 23%	42/83 51%	No Data	7/7 100%
Periapical lesion	1/40 60%	15/83 18%	5/109 71%	5/7 71%
Enamel hypoplastic defects	24/40 60%	48/83 58%	No Data	6/7 86%

**Note**. Cross Bones Burial Ground in Southwark, London, UK. Cadia Cemetery, NSW, Australia. St John’s, Milton, Otago, New Zealand. Further information regarding the comparative samples and the systems and methods used to categories/ collect the data is published in Gurr et al. 2023 [46]

Dental developmental defects of the enamel and dentine, e.g., enamel hypoplasia and interglobular dentine, seen in many of the St Mary’s non-adults and adults confirmed that these individuals had suffered but survived one or more insult to their health during the development of their dentition (Tables 6 and 12). Whether these conditions occurred before migration in the UK, during the voyage, or in SA is unknown. However, an adult with dental developmental defects could have been a child during the voyage to SA and have suffered a serious health insult (Tables 6 and 12) [46].

Evidence of the skeletal developmental defect sacral spina bifida occulta was seen in several individuals from St Mary’s (Table 6). This condition is compatible with a lack of folic acid in the mother’s diet during pregnancy (Vitamin B9), which leads to the incomplete closure of posterior vertebral arches of the sacrum [122]. This condition and other metabolic deficiencies, e.g., lack of Vitamin C and/ or iron (Table 6), may have occurred due to limited access to necessary nutrients, education and/ or poor antenatal health care. These conditions highlight some of the many challenges that the migrants faced.

Morphological changes to the bones due to diseases and degenerative conditions such as vertebral osteophytes, Schmorl’s nodes, and eburnation of vertebral facets and/or other surfaces were identified in eight adults from this excavated sample (Table 6) [45,47]. Bony outgrowths and/or changes to the structure of the joints would have caused pain and reduced the mobility of the sufferer and suggest that the affected individuals could had undertaken hard physical labour over a long period of time. Changes to the bone/s due to infectious disease for example tuberculosis, was also identified in the excavated skeletal remains from St Mary’s (Table 6) [43]. All these factors, such as poor oral health, diseases and deficiencies, and the demands of hard physical labour would have contributed to poor general health of this group.

### 2. Ships records

#### 2.1. The Voyages – UK to SA.

The number of voyages from the UK to SA between 1836–1885 were influenced by multiple interacting factors including political instability, economic fluctuations and environmental events (Fig 1). A dip in the number of ships arriving in SA were linked to the colony’s first economic depression, the multiple changes of colonial Governors and periods of drought (Fig 3) [20,38,123,124]. Increases in immigration trends were also closely tied to the colony’s evolving needs, for instance tradesmen, engineers, and builders were required to support infrastructure growth and development projects, whereas farm labours were needed to develop agricultural industries. Economic booms and crises prompted or reduced the number of emigrant voyages to the colony. British colonial policy, trade dynamics, and changes to the UK labour market could also influence the recruitment of migrants.

#### 2.2. Superintendent Surgeon and Documented Causes of Death at Sea.

Large emigrant ships traveling from the UK to SA could convey between 500–700 passengers at one time. This high number of people would mean there was limited space on board, and that disease could quickly spread through a vessel [100,101]. The cramped conditions, quality of nutrition and sanitation on the ship, as well as the competency of the Captain, Surgeon and the crew would have all impacted the health status of the passengers, with some of the early voyages having a high mortality rate (Tables 8 and 9).

The system of appointing a Superintendent Surgeon to each emigrant ship carrying more than 100 passengers aimed to reduce mortality rates at sea [42]. Excessive deaths during the voyage meant not only financial penalties for the surgeon but also risked damaging his medical reputation and that of the Captain and British Government. High mortality rates could even deter future migrants from traveling to SA, with the effect of potentially reducing political and economic gains.

Over time, the initial anticipated role of the Superintendent Surgeon evolved with emergent duties that encompassed numerous concerns and was not limited to attending sick and injured passengers. Dr George Mayo on board the emigrant ship ‘Asia’ [42,125,126] was called upon to settle a dispute between passengers. He notes, “This morning, I was called to Mr Letts whom Mr. C. Olliver had struck and made his face bleed and as the complaint came it was done without provocation. I had the parties into the cabin before myself and the captain…” [125]. He also recorded concerns about the migrants attitude, their food provisions and health in his diary, “I have great trouble to get the emigrants on deck” and “The rotten potatoes in the hold I think occasioned dysentery amongst the emigrants, spoken to Captain. Freeman.” [125]. These multiple duties are illustrative of non-predictive outcomes, both positive and negative and are consistent with a CAS framework.

The pattern of passenger deaths at sea by year (Figs 4 and 5) did not closely follow that of the number of voyages per year (Fig3) e.g., the years with the highest number of voyages (1849 and 1850) did not have the highest number of deaths. This suggests that other factors influenced the death rate at sea. On occasion, particular conditions such as cholera could affect a large number of the migrants during a voyage (Table 10) [100,101,127]. It is possible that these deaths were linked to similar epidemics on land such as the Broad Street cholera outbreak in London during the same year [103,128,129]. Contaminated water may have been brought aboard this ship. While the limits of this data do not give a clear answer, many deaths recorded as caused by cholera did occur in 1854 (Table 10) [103,126,129]. There may have been concern that ships affected by such conditions arriving in SA would affect the population of the new colony. However, available SA newspaper accounts relating to voyages with recorded cases of cholera suggest no active cases disembarked and that cases with this condition had previously been diagnosed in the colony [130–133].

The age and health status of an individual at the time of the voyage to SA meant that any infectious or non-infections conditions encountered on the ships could develop into fatal illnesses. Conditions such as measles, whooping cough, and/ or lack of cleanliness, and at times, an insufficient supply of medicines on board the ship were recorded by the ship’s surgeon (Fig 5) [100,101,103]. Other risks to the health and safety of the passengers during the voyage were connected to inadequate ventilation below decks, and flooding of their quarters due to severe weather conditions. At times, passengers in steerage (i.e., of the lower decks) may have been restricted to their quarters. Shipwreck was also a possibility [134].

A cause of death recorded in the ship’s log could have occurred due to multiple interacting conditions. The diagnoses recorded such as diarrhoea and/or fever are symptoms rather than diseases; and may be indications of one or more underlying condition. Diarrhoea was recorded as a major cause of death for non-adults by Superintendent Surgeons between 1849 and 1865 (Table 9) [135]. These individuals under the age of 4 years represented the largest demographic on the voyage and the most vulnerable age groups due to their developing immune system and the reduction of the maternal antibodies that had protected them during their first year of life. For many adults the major causes of death at sea were fever and ‘Phthisis’ a term used in the 19^th^ century for Tuberculosis [136] (Table 9). It is possible that some migrants were suffering from such medical conditions before embarking, from which they subsequently died at sea [135].

The death of many young children at sea and the conditions on the ships affecting the health of passengers could have had a multi-generational impact on the forming colony in South Australia. Outbreaks of disease at sea that were not fatal for many passengers, both non-adults and adults, may still have had lasting health impacts. For example, the health of a pregnant woman not only directly affected the unborn child, but also the child’s development and long-term health as an adult [137–144]. The number of deaths on emigrant ships decreased over time suggesting an improvement of the conditions on board. A change in the storage of food and water, and a growing awareness of how diseases were transmitted could have improved the health of the passengers, especially the most vulnerable such as infants, young children and pregnant women (Fig 4. and Table 7) [41,145].

The reduction in mortality on board could also be related to amendments to the Passenger Act of 1835, as well as technological advances such as ventilation machines for the lower decks of the ships and distillation of water [128,146,147]. Each of these factors can be understood as emergent societal structures (economic, political and ideological; see top of Fig 1) resulting from micro-level interactions in multiple voyages in the preceding years.

#### 3. Life in the new Colony.

While some migrants overcame the challenges and flourished in the new Province of South Australia there were others who had difficulties [33,148]. Complex dynamic interests that interacted to influence the colony’s development (Fig 1) could have also influenced and affected the health of the population [20,38,124].

The arrival of emigrant ships with large numbers of passengers should have provided the colony with a varied work force, but the time delay involved in communicating between Adelaide and London often led to the arrival of substantial numbers of immigrants all with similar skills for whom there was no employment [149] (see orange dashed line in Fig 3 showing accumulation of population based on arriving passengers). This could be due to the rapidly changing economy and added further to health and socioeconomic pressures. For example, records show that several groups of unmarried Irish women and girls arrived in SA, between 1848–1856, perhaps with a promise of work ‘in service’ as a maid or housekeeper and a future far from famine [102,150–152]. Their arrival, over a short period of time flooded the local labour market for domestic servants [152].

This unsustainable situation created by increased unemployment in the colony corresponds with a rise in the number of people seeking financial support from the Government and charitable organisations [153–156] and is a clear example of a macro-level emergent property (Fig 1). Official government returns for 1853 state that 464 people received relief from the Destitute Asylum, while in 1854 it had increased to 685. By 1855, this number was 3,027 [123,157]. This increase in unemployment and a need for charitable support coincided with the higher number of burials in the unmarked graves in the St. Mary’s Cemetery compared to later years.

As the colony matured a Constitution for South Australia was developed and elections held for a new Parliament [158–160]. This enabled some of the populace to vote and the colony to exercise self-rule. A change in the ‘Regulations for the selection of Persons in Britain for Free Passage’ to the colony in 1858, reduced the number of emigrants arriving from the UK (Fig 3) [160,161]. However, South Australia was now starting to generate its own future workforce. These changes in the demographic profile of the population are seen in the returns for the SA census of 1860 which confirms that one third of the population had now been born in the colony [162]. The newly formed Parliament of South Australia was also exploring the issue of social security through the Destitute Person’s Relief Act of 1866/ 67 [163], and the advance of tertiary education with the foundation of the University of Adelaide in 1874.

#### 3.1. St Mary’s burial records.

A similar pattern for the cause of deaths of the infants and younger children at sea and on land (St Mary’s) was identified (Tables 9–11). Atrophy/ marasmus and gastrointestinal conditions were commonly recorded for these age groups. Table 11 highlights that a ‘cause of death’ was often not recorded especially for the infants under one year of age in the St Mary’s documents. For the adults, at sea commonly recorded causes of death were fever, diarrhoea/ dysentery, cholera, pulmonary conditions (phthisis/ bronchitis/ pneumonia) and typhus. This is in contrast to adults listed in St Mary’s records where the most recorded causes of death were pulmonary conditions, general decay/ old age, conditions associated with the liver, kidneys and or heart and accidents.

These similarities in causes of death for the infants and young children confirmed their vulnerability to infections while their immune systems were developing, and they had a reduced ability to cope with dehydration due to their small body size. This is an example of how both similar (non-adults) and different (adults) outcomes can result in the same contexts based on the interaction of physiological and developmental factors and can be explained with a CAS understanding (Fig 1).

The limitations of each individual data source have been addressed above in each of the sections in which they are discussed. However, we argue that a CAS understanding deals with some of these limitations by providing a framework to combine evidence and explore interactions between different sources, which represent outcomes. An example is an adult whose skeleton showed traumatic injuries (fractures of the skull and jaw). The St Mary’s burial records list individuals who had suffered fatal injuries including the head. Coroner’s records and contemporary newspaper reports also recorded individuals who received such injuries. Future developments would explore this and similar examples by expanding the number and variety of data traces but following the same analysis and interpretative CAS framework.

## Conclusion

The Complex Adaptive Systems approach increases knowledge of the general health of 19^th^ century migrants from the UK to SA and results in additional insights that could not have been gained using a single analytical methodology.

This methodology provides a deeper understanding of the emergent and unpredicted outcomes that occur with the interaction of multiple factors derived from varied data sources related to a rare colonial South Australian skeletal sample and diverse historical texts. This study demonstrates that a multidisciplinary/ multi-methodological/ CAS approach and analysis can now be undertaken to explore the identities and life histories of specific individuals in this sample. It can be further developed and applied to other archaeological and modern samples.
